# Sensing Properties of Multiwalled Carbon Nanotubes Grown in MW Plasma Torch: Electronic and Electrochemical Behavior, Gas Sensing, Field Emission, IR Absorption

**DOI:** 10.3390/s150202644

**Published:** 2015-01-26

**Authors:** Petra Majzlíková, Jiří Sedláček, Jan Prášek, Jan Pekárek, Vojtěch Svatoš, Alexander G. Bannov, Ondřej Jašek, Petr Synek, Marek Eliáš, Lenka Zajíčková, Jaromír Hubálek

**Affiliations:** 1 Central European Institute of Technology, Brno University of Technology, Technická 3058/10, CZ-61600 Brno, Czech Republic; E-Mails: businova@feec.vutbr.cz (P.M.); xsedla44@stud.feec.vutbr.cz (J.S.); prasek@feec.vutbr.cz (J.P.); pekarek@feec.vutbr.cz (J.P.); vojtech.svatos@ceitec.vutbr.cz (V.S.); 2 Centre of Sensors, Information and Communication Systems, Faculty of Electrical Engineering and Communication, Technická 3058/10, CZ-61600 Brno, Czech Republic; 3 Central European Institute of Technology, Masaryk University, Kamenice 5, CZ-62500 Brno, Czech Republic; E-Mails: 234860@mail.muni.cz (A.G.B.); jasek@physics.muni.cz (O.J.); synek@physics.muni.cz (P.S.); mareke@physics.muni.cz (M.E.); lenkaz@physics.muni.cz (L.Z.); 4 Department of Physical Electronics, Faculty of Science, Masaryk University, Kotlářská 2, CZ-61137 Brno, Czech Republic

**Keywords:** carbon nanotubes, microwave torch, plasma enhanced chemical vapor deposition, electronic properties, electrochemical sensor, gas sensor, field emission, IR absorption

## Abstract

Vertically aligned multi-walled carbon nanotubes (VA-MWCNTs) with an average diameter below 80 nm and a thickness of the uniform VA-MWCNT layer of about 16 μm were grown in microwave plasma torch and tested for selected functional properties. IR absorption important for a construction of bolometers was studied by Fourier transform infrared spectroscopy. Basic electrochemical characterization was performed by cyclic voltammetry. Comparing the obtained results with the standard or MWCNT‐modified screen-printed electrodes, the prepared VA-MWCNT electrodes indicated their high potential for the construction of electrochemical sensors. Resistive CNT gas sensor revealed a good sensitivity to ammonia taking into account room temperature operation. Field emission detected from CNTs was suitable for the pressure sensing application based on the measurement of emission current in the diode structure with bending diaphragm. The advantages of microwave plasma torch growth of CNTs, *i.e.*, fast processing and versatility of the process, can be therefore fully exploited for the integration of surface-bound grown CNTs into various sensing structures.

## 1. Introduction

Carbon nanotubes (CNTs), a synthetic carbon allotrope, are made of sp^2^ hybridized carbon atoms. Single-walled CNT (SWCNT) is a graphene sheet rolled-up into a seamless cylinder and multi-walled CNT (MWCNT) is composed of several such cylinders, nested concentrically [1,2]. CNTs are often synthesized by a chemical vapor deposition (CVD) in the presence of a catalyst, nanoparticles of transition metals. In thermal CVD, a carbon-containing gas mixture is heated typically to 550–1100 °C by a conventional heat source. Plasma enhanced CVD (PECVD) activates the gas mixture by ignition of plasma discharge but a separate heating of the substrate might be required too [3]. General arguments for the PECVD include low-temperature, easily achieved vertical alignment and large area processing [4].The CVD methods are used for a CNT volume-synthesis and a surface-bound growth of vertically aligned and micropatterned CNT arrays [5]. The vertically-aligned CNTs (VA-CNTs) are highly desirable for the integration into functional devices. The aligned growth can produce CNTs free from amorphous carbon with a very narrow range of tube lengths and diameters, which is an additional advantage for many applications [6].

Unique physical and electrical properties of CNTs, *i.e.*, high electrical conductivity, remarkable mechanical strength, and thermal and chemical stability, predestinate them for many nanotechnology-based applications in electronic, optical and biomedical devices, sensors and composites [7,8]. The application potential of CNTs depends on their properties that are given by their structure and the form in which they are applied. Separated MWCNTs exhibited a non-linearity in I-V characteristics that were relatively large in case of the contacts made by underlying gold electrodes but almost diminished if the contacts were placed on the top of partially etched nanotubes [9]. Theoretically predicted impedance of SWCNT bundles at high frequencies is quite complex, employing resistance R, inductance L and capacitance C, and their validation proved to be difficult [10]. The experimentally proposed circuit model of SWCNTs contains a parallel RC element resulting from the contacts in series with R and L representing the SWCNT intrinsic behavior [10,11]. Similarly, an equivalent circuit model consisting of RC networks is constructed to simulate the electrical responses of MWCNT/polymer composites [12,13].

The optical properties of CNTs help to understand their structure [14], evaluate their purity [15] and open new applications for optical sensing [16,17]. MWCNT-based infrared detectors have received much attention due to MWCNT band gap of 0.4–6.0 eV and high absorption efficiency in IR [18]. A bolometer based on CNT/polymer composites was constructed for the detection of infrared radiation from the range 0.2–20 μm [19] and it was shown that the sensitivity and response time of the CNT-based bolometers can be substantially improved by an appropriate functionalization and selection of organic matrix [19,20].

CNTs exhibit also a great potential for electrochemical sensing due to their unique electrical properties, high surface area, fast heterogeneous electron transfer, and electrochemical stability [21–25]. The CNTs implemented as a VA-CNT film provide other advantages such as a controlled growth in defined areas and an easy modification of their surface demanded for particular sensing or biosensing applications [6]. The as-prepared VA-CNT-based sensors have been successfully applied to detect rutin [26] and salbutamol [27], a prohibited drug in sports. The VA-CNT thin films have also been tested as candidate platforms for DNA immobilization and detection of DNA hybridization [28]. The VA-CNT electrode modified by gold nanoparticles has been used for non-enzymatic detection of uric acid [29] and a platinum nanoparticle-modified VA-CNT electrode for detection of L-cysteine [30].

The CNTs belong to a group of new materials that have been extensively tested for gas sensing during the last 10 years and much effort has been put into the development of gas sensors working at room temperature. In spite of many papers devoted to SWCNT-based gas sensors [31–33], the MWCNTs can be also successfully used [34–36] and are preferable because of their low costs. The construction of room temperature ammonia (NH_3_) sensors is an important task because NH_3_ is a dangerous gas having a negative influence on the environment and humans. Cui *et al.* created a sensor with MWCNTs decorated by Ag nanocrystals that exhibited an enhanced response of 9% to 10,000 ppm of NH_3_ as well as fast recovery [37]. A high sensitivity—6.2% to 4000 ppm of NH_3_—was proven for Co_1−x_Ni_x_Fe_2_O_4_/MWCNT composites [38]. Varghese *et al.* investigated sensitivity of resistive and capacitive MWCNT sensors for different gases such as water vapors, NH_3_, CO_2_ and CO [39].

CNTs have been also considered as promising field emitters due to their low turn-on filed, long emitter lifetime and good emission stability [40]. The field-emitter configuration should have the highest aspect ratio and low work function at its surface. The first extensive study of field emission from CNTs has been published by Bonard *et al.* in 1998 [41]. Later on, CNTs were used as field-emitters in flat-panel field-emission displays [42] or electron sources in electron microscopes [43].

In the present work, a promising application potential of VA-MWCNTs grown by PECVD in microwave (MW) plasma torch [44,45] is explored in detail. Previous studies of the CNTs deposited by the MW plasma torch revealed possible improvements of the process [46] and provided a basic structural characterization of the CNTs using scanning and transmission electron microscopies and Raman spectroscopy [47]. The technology based on the MW torch is a high speed process that takes only 60–120 s including catalyst activation (restructuralization of a catalytic thin film into nanoparticles) and does not require any external heating source of the substrate. Besides starting the CNT growth with a thin catalytic film deposited on the substrate in a separate technological step, it offers the possibility to prepare catalytic nanoparticles during the same process, *i.e.*, using the MW plasma torch [48]. Another advantage is a successful preparation of vertically aligned MWCNTs directly on Si without using a barrier SiO_2_ layer [3]. A direct contact between the VA-CNTs and the Si substrate can be very important for some applications and it enables the construction of a field-emission based pressure sensor [49]. Besides the field emission, other functional properties (electrical and electrochemical, gas sensing, IR absorption) of the CNTs from the MW plasma torch are tested. Thus, the present work investigates and summarizes the CNT properties directly related to particular sensing applications and in some cases (field-emission pressure sensor, electrochemical sensor, gas sensor) describes the sensor structure and its preparation.

## 2. Experimental Section

### 2.1. CNT Deposition and Structural Characterization

The VA-MWCNT layers were deposited from Ar/CH_4_/H_2_ mixtures using the atmospheric pressure MW plasma torch operated at the power of 210 W. The flow rates of Ar, CH_4_ and H_2_ were Q_Ar_ = 700 sccm, Q_CH4_ = 19–38 sccm and Q_H2_ = 250 sccm, respectively. The substrate for the CNT growth was heated by the interaction with plasma, and its temperature (950–1050 K) was regulated by the distance from the plasma torch nozzle. The CNT growth time, that included also the catalyst activation phase, varied from 60 to120 s.

The aim of the present work was to investigate functional properties of the CNTs deposited by MW torch and, in some cases, integrate them into sensing devices. Most of the measurement structures were based on Si due to its compatibility with microelectronic chips and microelectromechanical systems (MEMS). CNTs were grown either on a polished single crystal silicon (c-Si) substrate, the c-Si coated with an adhesive metallic interlayer or the c-Si coated with in a thermal silicon oxide (SiO_2_) film. The latter is used if the application requires a dielectric or thermal separation of the CNTs. The thickness of SiO_2_ film did not play a significant role and was chosen arbitrarily. The substrate details are given in the following sections describing each particular measurement structure.

A vacuum evaporated Fe film, 5 nm in thickness, or Fe nanoparticles (NPs) deposited on the substrate by MW torch from iron pentacarbonyl (Fe(CO)_5_) vapors were used as catalysts. The details of the NPs deposition are described in previous papers [50,51]. The Ar flow through the central torch nozzle and through the blower with liquid Fe(CO)_5_ were 700 and 28 sccm, respectively. The Ar flow of 28 sccm through the blower with the liquid corresponds to 0.1 sccm of Fe(CO)_5_. The deposition time was 10 s.

Surfaces and cross sections of the prepared CNT samples were checked by the Tescan MIRA II LMU scanning electron microscope (SEM, TESCAN, Brno, Czech Republic) using 15 kV acceleration voltage.

### 2.2. Characterization of Electrical Behavior of CNTs

The CNTs for electrical characterization, such as I-V characteristics and sheet resistance measurements, were grown in the MW plasma torch on p-type, boron-doped, c-Si substrates (8 mm × 8 mm, thickness 525 μm, resistivity <6 Ω·cm^−1^) coated by the SiO_2_ film, 300 nm in thickness, and the top Fe catalytic layer. The square chips fully covered with CNT films were finished by vacuum evaporation of gold pads (0.5 mm × 0.5 mm) in each sample corner.

The chips were investigated at the probe station Cascade M150 connected to the Keithley SCS-4200 semiconductor analyzer (Keithley Instruments, Inc., Cleveland, OH, USA). The I-V characteristics of the films were measured between the opposite corners in the voltage range from −5 V to +5 V. The specific electrical resistance was determined by Van der Pauw measurement which was carried out automatically by the Keithley SCS-4200 analyzer.

For the impedance spectroscopy, the CNTs were grown on a SITAL glass-ceramic substrate (10 mm × 15 mm, thickness 525 μm) using Fe catalytic layer. This substrate was chosen to suppress the effect of Si substrate properties during the impedance spectroscopy measurement. The VA-MWCNTs were deposited on a central circular area, 6 mm in diameter. Two circular gold contacts of 3 mm in diameter were prepared by vacuum evaporation. The impedance was measured using the Agilent E4980A Precision LCR meter (Agilent Technologies, Santa Clara, CA, USA) and data acquisition by LabView software (National Instruments, Austin, TX, USA). The measurements were performed in the frequency range from 20 Hz to 2 MHz with the voltage level of 0.5 V.

### 2.3. IR Absorption

The FTIR measurement procedure is a simple way to compare samples with or without grown CNTs in order to consider CNTs as possible IR detector. The CNTs for infrared absorption measurements were grown on the same substrates as described in the previous Section 2.2, *i.e.*, c-Si substrate with the SiO_2_/Fe double layer. In the case of the bolometer, thin SiO_2_ layer is needed for construction of low thickness MEMS diaphragm to suppress thermal loses to the substrate mass [52].

The IR absorption of VA-CNTs on the Si substrate covered by 300 nm thick SiO_2_ film was investigated with the Fourier transform infrared (FTIR) spectrometer Nicolet iS50 in the attenuated total reflection (ATR) mode. The ATR crystal was pressed against the sample and the measurement was performed in the wavelength range 2.5–22.5 μm. The absorbance of the VA-CNT sample was compared to the absorbance of Si substrate covered by 300 nm thick SiO_2_ film and 5 nm thick Fe film used as catalyst of the CNT growth.

### 2.4. Electrochemical Characterization

The preparation of samples for the electrochemical measurements started with n-type, highly antimony-doped, c-Si substrates (5 mm × 30 mm, thickness 525 μm, resistivity <0.02 Ω·cm^−1^) coated by the SiO_2_ film, 300 nm in thickness. The working electrode had dimensions 4.5 mm × 4.5 mm and consisted of the CNTs deposited in the MW plasma torch (Section 2.1). Before the CNT growth, the SiO_2_ insulating film was removed from the working electrode area by a wet chemical etching in buffered HF and the area of the working electrode was covered by magnetron sputtered Ti (10 nm)/Ta (250 nm) double layer and vacuum evaporated top Fe film, the catalyst for the CNT growth. The Ti/Ta coating was necessary to ensure a good adhesion of the CNTs that otherwise peeled off the substrate when immersed in an electrolyte. The highly doped Si substrate was needed for a good electrical connection between the CNT layer and the contact pads which were situated on the opposite end of the substrate with the same dimensions as the electrode.

Electrochemical response of the [Fe(CN)_6_]^4−/3−^ redox couple mediated by the CNT electrode was investigated by the cyclic voltammetry (CV) with AUTOLAB PGSTAT 204 potentiostat/galvanostat controlled by Nova 1.10 software (Metrohm Autolab B.V., Utrecht, The Netherlands). A standard three-electrode voltammetric cell employing an Ag/AgCl reference electrode (type 6.0729.100, Metrohm, Herisau, Switzerland) and a platinum auxiliary electrode (type 6.0343.000, Metrohm) was used for all the experiments. The electrolyte was an equimolar solution of 2.5 mM potassium ferrocyanide and potassium ferricyanide ([Fe(CN)_6_]^4−/3−^) in 0.1 M KCl. The cyclic voltammograms were recorded in the potential range from −1 V to +1 V with scan rates (*υ*) from 5 to500 mV·s^−1^.

### 2.5. Gas Sensing Properties

The CNTs for testing the gas sensing properties were prepared on the p-type, boron-doped, c-Si substrates (8 mm × 8 mm, thickness 525 μm, resistivity <6 Ω·cm^−1^) coated with the 92 nm thick SiO_2_ film. The catalytic Fe nanoparticles were deposited by the MW plasma torch (see Section 2.1) in the central area, 4 mm × 4 mm, of the substrate. This form of the Fe catalyst was chosen for the growth of a less dense CNT mesh because the gas sensing application requires a large surface area and dense CNTs mask each other. The CNTs were grown from Fe NPs in the MW plasma torch (Ar = 700 sccm, H_2_ = 250 sccm, CH_4_ = 38 sccm, deposition time 60 s, deposition temperature 973 K) as described in Section 2.1. The measurement chip was finished by vacuum evaporated gold contacts (15 nm NiCr adhesion layer with 350 nm Au layer on the top) with a size of 2 mm × 6 mm centered symmetrically to the middle of the sensor.

The gas sensing properties were determined as a change of the sample resistance during its exposure to a gaseous analyte, either ammonia (NH_3_) or isobutane (iC_4_H_10_). The measurements were performed in a custom-built gas station equipped with two gas channels and one chamber for two sensors' characterization at once. One gas channel is used for the synthetic air as a carrier gas. The second gas channel supplies diluted analytes, NH_3_ or iC_4_H_10_ in nitrogen. The response of the sensors was determined at different analyte concentrations, namely 100 ppm, 250 ppm and 500 ppm. Before each measurement, a sample conditioning was carried out for 30 min at 200 °C in the air flow of 1000 sccm. The sensor response was defined as

(1) $$\Delta {\text{R}/\text{R}}_{0}=\left(\left({\text{R}–\text{R}}_{0}\right){/\text{R}}_{0}\right)\times 100\%$$

where R is the resistance of the sensor exposed to the analyte and R_0_ is the sensor resistance in pure air. The sensitivity tests were performed at two temperatures, room temperature and 200 °C. The resistance R_0_ was determined from 60 min measurement in air flow of 500 sccm. The sensor response to analyte was measured as three 10 min cycles (for 100 ppm, 250 ppm and 500 ppm of analyte) alternated with three 10 min cycles in air flow. The total gas flow rate was kept constant at 500 sccm.

### 2.6. Field Emission Properties for Pressure Sensing

**Figure 1.**
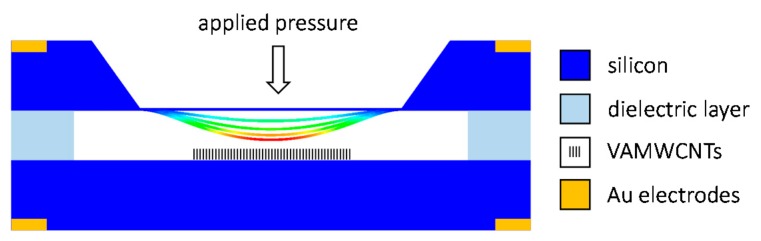
Schematic view of the MEMS pressure sensor with carbon nanotubes emitters.

The MEMS field emission pressure sensor was designed as a diode structure (see Figure 1). It consisted of two n-type, highly antimony-doped, c-Si electrodes (10 mm × 15 mm, thickness 525 μm, resistivity <0.02 Ω·cm^−1^). One of them was anisotropically etched to a bending diaphragm. The other was coated by an emissive material, the VA-MWCNTs deposited in the MW plasma torch (see Section 2.1). The highly doped Si substrate was needed for a good electrical connection between the CNT layer and the contact pads. The Fe catalytic film, required for the growth of CNTs, was deposited in the center of the substrate on the area of 4 mm × 4 mm. A native oxide film on Si was removed by HF prior to the deposition of the Fe film, thus ensuring the electrical contact between CNTs and Si. The field-emission pressure sensor is proposed to be constructed by the separation of the electrodes with a dielectric layer creating an integrated evacuated volume. The dielectric layer can be made of Pyrex or Simax glass using anodic bonding technology or made of glass frit using a screen printing process.

The measurements of the field emission properties were carried out in a vacuum chamber at pressure lower than 10^−4^ Pa. A diaphragm bending was simulated by the linear nano-motion drive SmarAct enabling precise changes of the distance between the two electrodes inside the vacuum chamber with the step from 50 nm to1000 nm. The initial distance of 120 μm was established using a solid dielectric foil that was then removed and the emitter-to-anode distance was set up with the SmarAct drive from 84 μm to120 μm. The measurement voltage from 0 to150 V was automatically applied using software communicating via GPIB with the voltage supply.

## 3. Results and Discussion

### 3.1. SEM of CNTs

**Figure 2.**
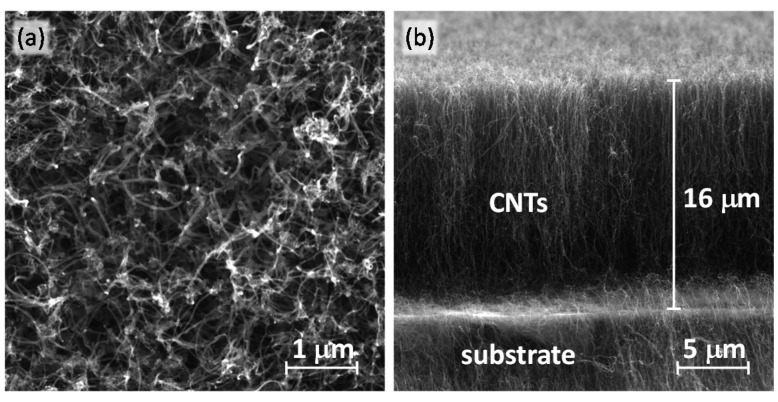
Typical SEM micrographs of the silicon substrate covered with VA-MWCNTs prepared in MW plasma torch (Ar = 700 sccm, H_2_ = 250 sccm, CH_4_ = 25 sccm, deposition time 60 s, deposition temperature 973 K): (**a**) top view of VA-MWCNTs and (**b**) cross-sectional view of the VA-MWCNT film.

A fast growth of VA-MWCNT films on the c-Si and c-Si/SiO_2_ substrates has been achieved in MW plasma torch operated at atmospheric pressure without an external heating source [44,45]. The characterization of the VA-MWCNT films by SEM, transmission electron microscopy (TEM), Raman spectroscopy and the influence of process parameters on the CNT growth were discussed in detail in our previous publications [3,46,47]. Typical SEM images of prepared VA-MWCNT film are shown in Figure 2. Although the cross-sectional view in Figure 2b confirms the vertical alignment of the CNT film, the top view (Figure 2a) reveals that the alignment at the end of nanotubes is not perfect. The CNTs having a high aspect ratio, less than 80 nm in the diameter and a length of about 16 μm, are curled at the top end due to different heights. Therefore, top view micrographs cannot provide sufficient information about the structure of all the CNT film.

### 3.2. Electrical Properties of CNTs

**Figure 3.**
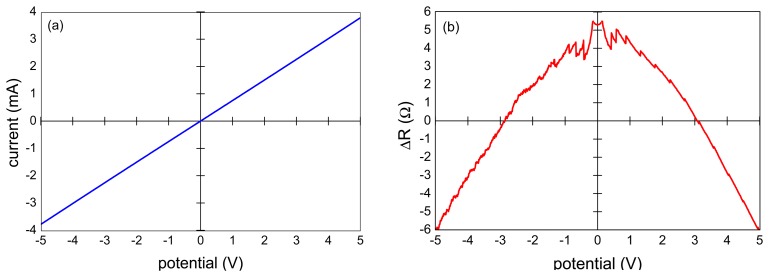
(**a**) I-V characteristic and (**b**) deviation of measured resistance from linear regression.

The measured I-V characteristics of the VA-MWCNT samples were nearly linear as documented in Figure 3a for the sample shown in Figure 2. The resistance was in the range of 1–1.3 kΩ. The specific electrical resistance was about 0.5 Ω·cm as calculated from Van der Pauw measurement and the film thickness of 16 μm determined by SEM. A small nonlinearity was revealed when the difference of the sample resistance and its linear approximation was plotted (Figure 3b). The deviation from linear behavior was governed by an exponential growth with a small exponent. The nonlinearity of the CNT resistance did not exceed 6 Ω which corresponded to 0.5% of the film resistance.

**Figure 4.**
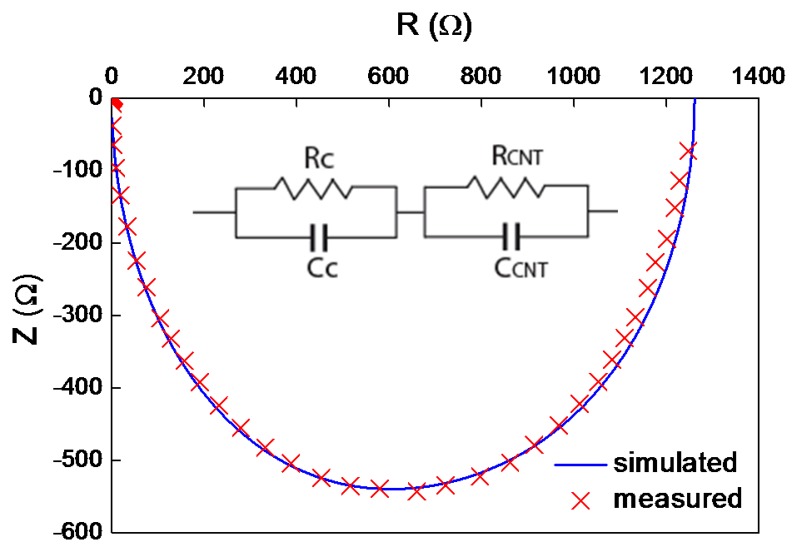
Impedance characteristics, crossed markers are measured data, dashed line is simulated according the inset equivalent circuit.

Fitting of the impedance measurements confirmed a simple RC model of two resistances and capacitances in parallel (Figure 4). According to Plombon *et al.* [10], the contact pads added a significant part of the RC circuit element. The R_C_/C_C_ circuit represents the contact impedance, and R_CNT_/C_CNT_ stands for the impedance of the vertically aligned CNT film. The resistances of the contact and the CNT films were 500 and 700 Ω, respectively. The capacitance of the contacts was much higher, about 15 nF, than the capacitance of the CNT film, 3.5 nF. It means that the surface of nanotubes is not pure enough to create a good contact. Adsorbed molecules such as water, CO_2_ and O_2_ can create a dielectric film that contributes to its high capacitance.

### 3.3. IR Absorption

**Figure 5.**
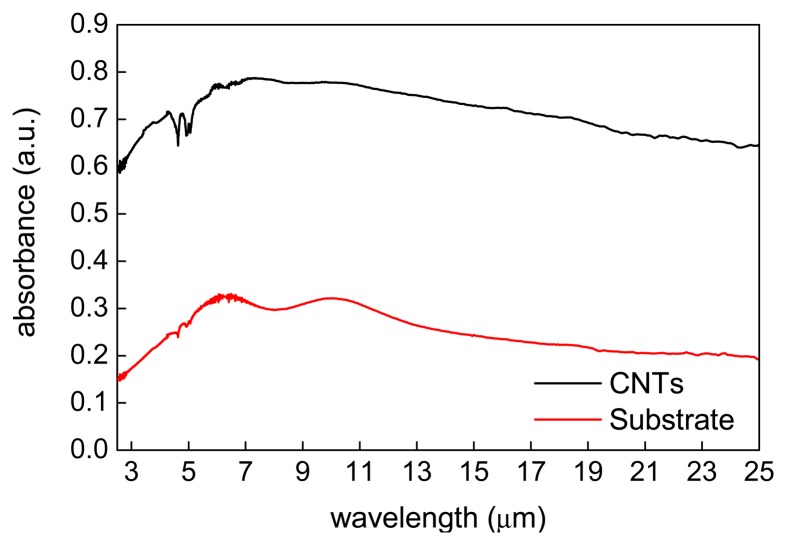
ATR-FTIR spectra obtained for the substrate with the catalytic layer (red line), and the CNT structure (black line).

The results of the ATR-FTIR study are shown in Figure 5. They indicate that the VA-MWCNTs can be affectionately applied as a possible absorption layer for IR detection. The mean absorbance value of nearly 80% was obtained. In Figure 5, the interval from 2.5 μm to 7.5 μm represents the absorbance of substrate. The local low points of the curve at approximately 9.0 μm show the typical progression for the atmospheric humidity. The absorbance of the CNTs is mostly seen in the interval from 8 μm to 22 μm which therefore includes the atmospheric window for IR detection.

From the physical principle of the material absorption, it has been well known the particle dipole moment is necessary. No modification is required for CNTs to create the dipole moment according to this measurement. The absorption in the IR region causes changes of vibrational and rotational status of the molecules. The absorption intensity depends on the IR photon energy which can be transferred to the molecule and this depends on the change of the dipole moment that occurs as a result of molecular vibration. As a consequence, a particle will absorb the IR light only if the absorption causes a change in the dipole moment. The absorption frequency is dependent on the vibrational frequency of the molecule.

### 3.4. Electrochemical Properties

**Figure 6.**
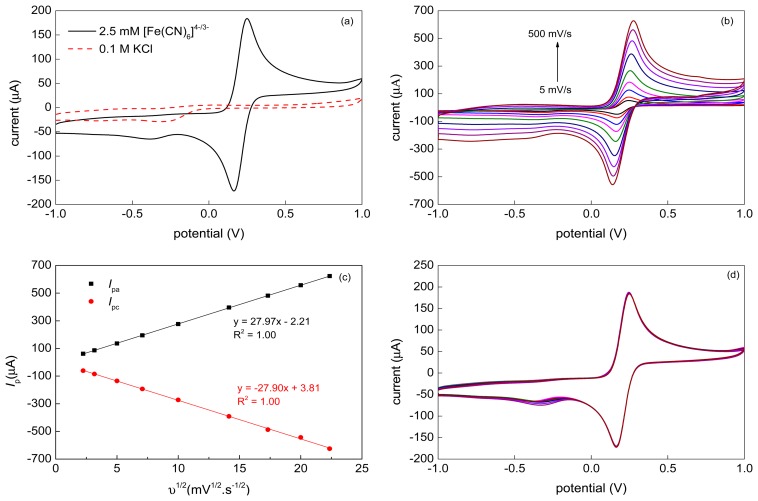
(**a**) Cyclic voltammograms obtained for 0.1 M KCl and 2.5 mM [Fe(CN)6]^4−/3−^ in 0.1 M KCl at *υ* = 50 mV·s^−1^ using VAMWCNT-based working electrode; (**b**) Cyclic voltammograms obtained for 2.5 mM [Fe(CN)6]^4−/3−^ in 0.1 M KCl at various scan rates (*υ* = 5, 10, 25, 50, 100, 200, 300, 400 and 500 mV·s^−1^) using VAMWCNT-based working electrode; (**c**) *I*_pa_*vs. υ*^1/2^ and *I*_pc_*vs. υ*^1/2^ curves; (**d**) 10 cycles of CV obtained in 2.5 mM [Fe(CN)6]^4−/3−^ in 0.1 M KCl at *υ* = 50 mV·s^−1^ using VAMWCNT-based working electrode.

A representative cyclic voltammogram recorded at 50 mV·s^−1^ with the VA-MWCNT electrode and 2.5 mM [Fe(CN)_6_]^4−/3−^ (1:1) solution in 0.1 M KCl is shown in Figure 6a. Two well-defined symmetric redox peaks separated by Δ*E*_p_ = (*E*_pa_ − *E*_pc_) = 83 mV were observed. The ratio of the anodic and cathodic peak currents reached unity (*I*_pa_/*I*_pc_ = 1.01). The results indicated that the VA-MWCNT electrode promote electron transfer quite well.

The effect of varying scan rates was studied for the scan rate range 5–500 mV·s^−1^. The corresponding cyclic voltammograms are given in Figure 6b. The anodic (*I*_pa_) and cathodic (*I*_pc_) peak currents varied linearly with the square root of the scan rate (*υ*^1/2^), as shown in Figure 6c. It demonstrates that the electrode process is controlled by a diffusion. The stability of the VA-MWCNT electrode was studied for the [Fe(CN)_6_]^4−/3−^ in 0.1 M KCl at 50 mV·s^−1^ using 10 cycles of CV. The results, depicted in Figure 6d, revealed that both, the oxidative and reductive, peak currents of the studied redox couple remained practically constant throughout all 10 potential cycles and, therefore, the CNT-based working electrodes with the Ti/Ta adhesive interlayers are suitable for repeated measurements.

Comparing the obtained results with the standard or MWCNT-modified screen-printed electrodes [53], the prepared VA-MWCNT electrodes indicate their high potential for construction of electrochemical sensors or biosensors detecting substances in aqueous solutions.

### 3.5. Gas Sensing Properties

**Figure 7.**
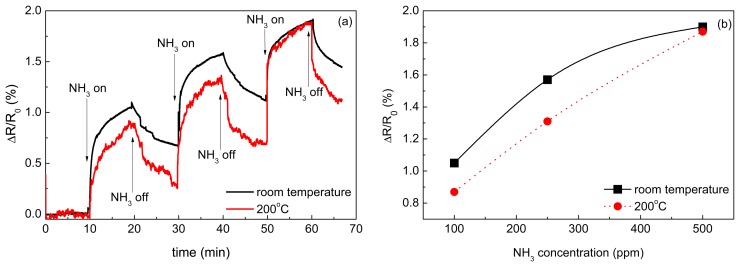
(**a**) Time dependent response of the CNT sensor to NH_3_ at room temperature and at 200 °C and (**b**) the response of the sensor determined after 10 min of the exposure as a function of NH_3_ concentration.

**Figure 8.**
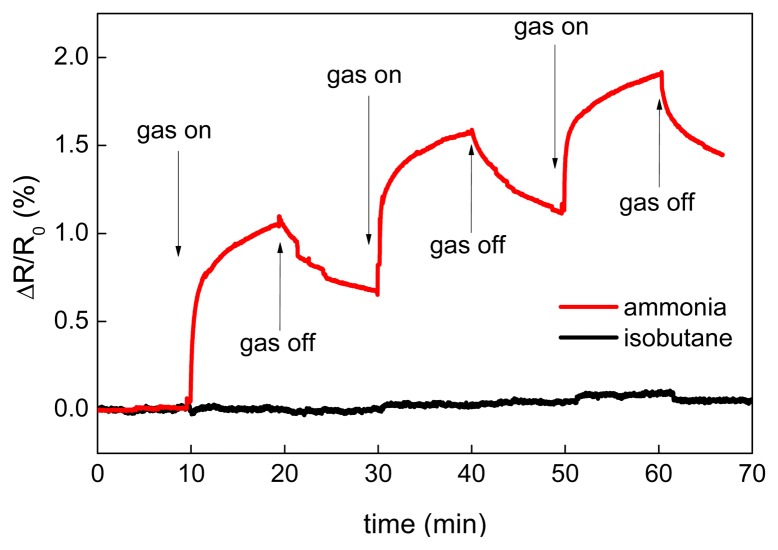
Time dependent response of the CNT sensor to NH_3_ and iC_4_H_10_ at room temperature.

The results of the CNT sensor response to NH_3_ at room temperatures and 200 °C are shown in Figure 7a. Since the sensor resistance increased during ammonia exposure, it is concluded that free carriers—the holes—in MWCNTs were neutralized by electrons coming from adsorbed NH_3_ and, therefore, MWCNTs exhibited p-type nature. This phenomenon has been already described in previous studies. Hoa *et al.* supposed that MWCNT is a p-type semiconductor and the adsorption of electron donor compound such as NH_3_ decreases the charge carrier concentration, thus inducing an increase of the resistance [54]. The same mechanism of interaction of adsorbed electron-donor compound with *p*-type CNTs was also described in [34,55–57]. The main interaction mechanism of ammonia with CNTs is a reversible adsorption. Vikramaditya *et al.* assume that NH_3_ is physisorbed on non-doped CNTs [58]. Thus, the sensing mechanism of ammonia with CNTs consists of two main steps, (i) physical adsorption of gas molecules and (ii) a charge transfer between adsorbed molecules and CNTs [59,60]. Testing of the sensor sensitivity to isobutane was carried out at room temperature (Figure 8) and revealed that the sensor is insensitive to a non‐polar molecule such as iC_4_H_10_.

According to the data presented in Figure 7b, the sensor exhibited higher response to NH_3_ at the room temperature than at 200 °C. Since the adsorption is an exothermal process, it is enhanced with the decreasing temperature. The response ΔR/R_0_ ranged from 0.87% to 1.9%. The dependence of the sensor response ΔR/R_0_ determined after 10 min of the exposure on the concentration of NH_3_ was almost linear at 200 °C whereas the relation became strongly nonlinear at room temperature. It can be linked to an incomplete desorption of NH_3_ at room temperature.

The obtained response of the sensors to NH_3_ was quite high taking into account that MWCNTs were not treated or modified for sensing. The highest response, 1.9%, was achieved at room temperature for 500 ppm of NH_3_. For comparison, Hoa *et al.* [54] achieved 8% response for 6% of NH_3_ in N_2_, *i.e.*, for 60,000 ppm. Here, the reported sensor was not tested for such a high concentration of NH_3_ but its response to a 120× lower concentration was only four times lower. A response of 9%, but to a lower concentration of 1% (10,000 ppm), was reported by Cui *et al.* for Ag-decorated MWCNTs [37].

In conclusion, the described sensor preparation technique is quite simple and allows achieving a sufficiently high response. The direct nanotube growth on the substrate allows managing the response by synthesis conditions that is critical for a scale-up production. The present CNT sensor can be further improved by a suitable functionalization and/or a decoration of the CNTs with metallic nanoparticles using the same set-up or a different procedure.

### 3.6. Field Emission Properties for Pressure Sensing

**Figure 9.**
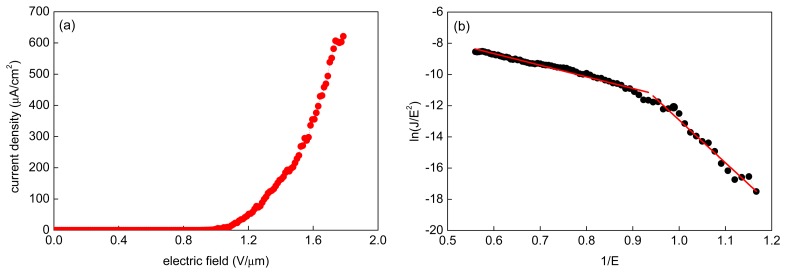
(**a**) Current density in dependence on field intensity for the CNT array field emitters; (**b**) The relevant Fowler‐Nordheim curve.

The measurements of field emission current were carried out multiple times at 10 emitter-to-anode distances from 84 μm to120 μm. The current density in dependence on the electric field intensity calculated from measured results is shown in Figure 9a and the corresponding Fowler-Nordheim (F-N) plot is in Figure 9b. Straight lines in the F-N plot indicates the quantum mechanical tunneling characteristic of the electron field emission and are based on the F-N equation

(2) $$I=(\text{A}\alpha \beta^{2}V^{2}/x^{2}\phi )\exp[-\text{B}\phi^{3/2}x/(\beta V)]$$

where *I* is the emission current, A = 1.56 × 10^−6^ A·V^−2^·eV, B = 6.83 × 10^9^ eV^−3/2^·V·m^−1^, *α* is the emission area, *β* is the field enhancement factor, *ϕ* is the work function, *x* is the distance between the anode and the emitter, and *V* is the applied voltage. Emission of many electrons at a low applied voltage requires a low work function (*ϕ*) and a high field enhancement factor (*β*). Using the F-N plot, *ln(J/E^2^) vs. 1/E*, the field enhancement factor can be determined from the slope of the straight line. The measured data can be divided into two sections, below and above 0.9 μm·V^−1^, that have different enhancement factors. Such behavior of the plot is not unusual and can be caused by various reasons, such as resistance, gas absorption, localized states or interaction between emitters [61,62].

The turn-on field, defined as the field intensity enabling the emission current density of 10 μA·cm^−2^, was determined to be below 1 V·μm^−1^. The current density of hundreds of μA was achieved already at 1.8 V·μm^−1^. The measured data follow the Fowler-Nordheim law in Equation (2) concerning the dependence of the emission current on the electrode distance when the applied voltage is fixed. Similarly, for the fixed distance between the electrodes, the emission current increases with increasing voltage. This confirms the expected behavior of our field emission electrode as proposed in the pressure sensor design. From the measured characteristics, one could also conclude that it is of advantage to operate at a higher electric field and shorter distances. In these conditions, a bigger change of the emission current with changing diaphragm deformation (pressure) leads to the higher sensitivity of the sensor, *i.e.*, higher Δ*I*/Δ*p* with the identical Δ*d*. This effect can be strengthened with the proper choice of diaphragm [49].

## 4. Conclusions

Selected functional properties of VA-MWCNT films were investigated in different types of measurement devices that integrated CNTs prepared in a microwave plasma torch. The average diameter of prepared MWCNTs was lower than 80 nm and the thickness of uniform VA-MWCNT layers was about 16 μm. Electrical measurements of CNTs showed their resistive behavior with a low resistance of 0.5 Ω·cm, and non-linearity of about 0.5%. A parallel capacitance proposed in previously published papers was confirmed by dynamic measurements. The FTIR analysis demonstrated a high absorption in the IR range of 8–22 μm allowing VA-MWCNT application in IR thermometers such as bolometers usually used in thermo-vision. Cyclic voltammetry using redox couple of [Fe(CN)_6_]^4−/3−^ were used to investigate the electrochemical response of the working electrodes coated by plasma grown VA-MWCNTs. The obtained results indicated good electrochemical properties (*I*_pa_/*I*_pc_ = 1.01, Δ*E*_p_ = 83 mV) and confirmed that studied VA-MWCNT working electrodes can be successfully used in the construction of electrochemical sensors.

The constructed resistive sensor based on VA-MWCNTs exhibited increasing resistance of the VA-MWCNT layer when exposed to NH_3_ that must be explained by the p-type semiconducting behavior of CNTs when adsorbed ammonia gas molecules donate electrons to CNTs. The field emission properties of the electrode with VA-MWCNTs grown directly on silicon were investigated with the aim to use the electrode in pressure sensing applications. The measured dependencies showed that emission current from the CNTs is stable, and a relatively low noise can be achieved for smaller electrode distances and higher voltage between the electrodes. The characteristics were reversible, with low turn-on field (1 V·μm^−1^), and current of hundreds of μA for the electric field was around 1.8 V·μm^−1^. It indicated that plasma-grown VA-MWCNTs are well suited for pressure sensing.

## References

[1] Meyyappan M. (2005). Carbon Nanotubes: Science and Applications.

[2] Backes C. (2012). Noncovalent Functionalization of Carbon Nanotubes: Fundamental Aspects of Dispersion and Separation in Water.

[3] Zajíčková L., Jašek O., Eliáš M., Synek P., Lazar L., Schneeweiss O., Hanzlíková R. (2010). Synthesis of carbon nanotubes by plasma-enhanced chemical vapor deposition in an atmospheric-pressure microwave torch. Pure Appl. Chem..

[4] Yen J.H., Leu I.C., Wu M.T., Lin C.C., Hon M.H. (2004). Density control for carbon nanotube arrays synthesized by ICP-CVD using AAO/Si as a nanotemplate. Electrochem. Solid State Lett..

[5] Prášek J., Drbohlavová J., Chomoucká J., Hubálek J., Jašek O., Adam V., Kizek R. (2011). Methods for carbon nanotubes synthesis-review. J. Mater. Chem..

[6] Chen H., Roy A., Baek J.B., Zhu L., Qu J., Dai L.M. (2010). Controlled growth and modification of vertically-aligned carbon nanotubes for multifunctional applications. Mater. Sci. Eng. R-Rep..

[7] Tsierkezos N.G., Szroeder P., Ritter U. (2011). Multi-walled carbon nanotubes as electrode materials for electrochemical studies of organometallic compounds in organic solvent media. Monatshefte Chem.-Chem. Mon..

[8] Tsierkezos N.G., Szroeder P., Ritter U. (2011). Application of Films Consisting of Carbon Nanoparticles for Electrochemical Detection of Redox Systems in Organic Solvent Media. Fuller. Nanotub. Carbon Nanostruct..

[9] Ahlskog M., Hakonen P., Paalanen M., Roschier L., Tarkiainen R. (2001). Multiwalled carbon nanotubes as building blocks in nanoelectronics. J. Low Temp. Phys..

[10] Plombon J.J., O'Brien K.P., Gstrein F., Dubin V.M., Jiao Y. (2007). High-frequency electrical properties of individual and bundled carbon nanotubes. Appl. Phys. Lett..

[11] Ksenevich V.K., Gorbachuk N.I., Poklonski N.A., Samuilov V.A., Kozlov M.E., Wieck A.D. (2012). Impedance of Single-Walled Carbon Nanotube Fibers. Fuller. Nanotub. Carbon Nanostruct..

[12] Geng S.N., Wang P., Ding T.H. (2011). Impedance characteristics and electrical modelling of multi-walled carbon nanotube/silicone rubber composites. Compos. Sci. Technol..

[13] Allaoui A., Hoa S.V., Pugh M.D. (2008). The electronic transport properties and microstructure of carbon nanofiber/epoxy composites. Compos. Sci. Technol..

[14] Popov V.N. (2004). Carbon nanotubes: Properties and application. Mater. Sci. Eng. R Rep..

[15] Goak J.C., Lee H.S., Han J.H., Park J.-Y., Seo Y., Kim K.B., Lee N. (2014). New metric for evaluating the purity of single-walled carbon nanotubes using ultraviolet–visible-near infrared absorption spectroscopy. Carbon.

[16] Kruss S., Hilmer A.J., Zhang J., Reuel N.F., Mu B., Strano M.S. (2013). Carbon nanotubes as optical biomedical sensors. Adv. Drug Deliv. Rev..

[17] Huang H., Zou M., Xu X., Liu F., Li N., Wang X. (2011). Near-infrared fluorescence spectroscopy of single-walled carbon nanotubes and its applications. TrAC Trends Anal. Chem..

[18] Gohier A., Dhar A., Gorintin L., Bondavalli P., Bonnassieux Y., Cojocaru C.S. (2011). All-printed infrared sensor based on multiwalled carbon nanotubes. Appl. Phys. Lett..

[19] Aliev A.E. (2008). Bolometric detector on the basis of single-wall carbon nanotube/polymer composite. Infrared Phys. Technol..

[20] Afrin R., Shah N.A., Abbas M., Amin M., Bhatti A.S. (2013). Design and analysis of functional multiwalled carbon nanotubes for infrared sensors. Sens. Actuators A Phys..

[21] Gao C., Guo Z., Liu J.-H., Huang X.-J. (2012). The new age of carbon nanotubes: An updated review of functionalized carbon nanotubes in electrochemical sensors. Nanoscale.

[22] Vashist S.K., Zheng D., Al-Rubeaan K., Luong J.H.T., Sheu F.-S. (2011). Advances in carbon nanotube based electrochemical sensors for bioanalytical applications. Biotechnol. Adv..

[23] Jacobs C.B., Peairs M.J., Venton B.J. (2010). Review: Carbon nanotube based electrochemical sensors for biomolecules. Anal. Chim. Acta.

[24] Ahammad A.J.S., Lee J.J., Rahman M.A. (2009). Electrochemical Sensors Based on Carbon Nanotubes. Sensors.

[25] Agüí L., Yáñez-Sedeño P., Pingarrón J.M. (2008). Role of carbon nanotubes in electroanalytical chemistry: A review. Anal. Chim. Acta.

[26] Ye M.L., Xu B., Zhang W.D. (2013). Voltammetric Behavior of Rutin at a Vertically Aligned Multiwalled Carbon Nanotubes Electrode. Sens. Lett..

[27] Karuwan C., Wisitsoraat A., Sappat A., Jaruwongrungsee K., Patthanasettakul V., Tuantranont A. (2010). Vertically Aligned Carbon Nanotube Based Electrochemcial Sensor for Salbutamol Detection. Sens. Lett..

[28] Berti F., Lozzi L., Palchetti I., Santucci S., Marrazza G. (2009). Aligned carbon nanotube thin films for DNA electrochemical sensing. Electrochim. Acta.

[29] Wang J.A., Zhang W.D. (2011). Sputtering deposition of gold nanoparticles onto vertically aligned carbon nanotubes for electroanalysis of uric acid. J. Electroanal. Chem..

[30] Ye M.L., Xu B., Zhang W.D. (2011). Sputtering deposition of Pt nanoparticles on vertically aligned multiwalled carbon nanotubes for sensing L-cysteine. Microchim. Acta.

[31] Feng X., Irle S., Witek H., Morokuma K., Vidic R., Borguet E. (2005). Sensitivity of ammonia interaction with single-walled carbon nanotube bundles to the presence of defect sites and functionalities. J. Am. Chem. Soc..

[32] Ndiaye A., Bonnet P., Pauly A., Dubois M., Brunet J., Varenne C., Guerin K., Lauron B. (2013). Noncovalent Functionalization of Single-Wall Carbon Nanotubes for the Elaboration of Gas Sensor Dedicated to BTX Type Gases: The Case of Toluene. J. Phys. Chem. C.

[33] Datta K., Ghosh P., More M.A., Shirsat M.D., Mulchandani A. (2012). Controlled functionalization of single-walled carbon nanotubes for enhanced ammonia sensing: A comparative study. J. Phys. D Appl. Phys..

[34] Zhou Y., Jiang Y.D., Xie G.Z., Du X.S., Tai H.L. (2014). Gas sensors based on multiple-walled carbon nanotubes-polyethylene oxide films for toluene vapor detection. Sens. Actuators B: Chem..

[35] Cava C.E., Salvatierra R.V., Alves D.C.B., Ferlauto A.S., Zarbin A.J.G., Roman L.S. (2012). Self-assembled films of multi-wall carbon nanotubes used in gas sensors to increase the sensitivity limit for oxygen detection. Carbon.

[36] Ahn K.S., Kim J.H., Lee K.N., Kim C.O., Hong J.P. (2004). Multi-wall carbon nanotubes as a high-efficiency gas sensor. J. Korean Phys. Soc..

[37] Cui S.M., Pu H.H., Lu G.H., Wen Z.H., Mattson E.C., Hirschmugl C., Gajdardziska-Josifovska M., Weinert M., Chen J.H. (2012). Fast and Selective Room-Temperature Ammonia Sensors Using Silver Nanocrystal-Functionalized Carbon Nanotubes. ACS Appl. Mater. Int..

[38] Tang Y., Zhang Q.H., Li Y.G., Wang H.Z. (2012). Highly selective ammonia sensors based on Co_1−x_Ni_x_Fe_2_O_4_/multi-walled carbon nanotubes nanocomposites. Sens. Actuators B Chem..

[39] Varghese O.K., Kichambre P.D., Gong D., Ong K.G., Dickey E.C., Grimes C.A. (2001). Gas sensing characteristics of multi-wall carbon nanotubes. Sens. Actuators B Chem..

[40] Wilfert S., Edelmann C. (2012). Field emitter-based vacuum sensors. Vacuum.

[41] Bonard J.M., Maier F., Stockli T., Chatelain A., de Heer W.A., Salvetat J.P., Forro L. (1998). Field emission properties of multiwalled carbon nanotubes. Ultramicroscopy.

[42] Guo P.S., Chen T., Chen Y.W., Zhang Z.J., Feng T., Wang L.L., Lin L.F., Sun Z., Zheng Z.H. (2008). Fabrication of field emission display prototype utilizing printed carbon nanotubes/nanofibers emitters. Solid State Electron..

[43] Nakahara H., Kusano Y., Kono T., Saito Y. (2009). Evaluations of carbon nanotube field emitters for electron microscopy. Appl. Surf. Sci..

[44] Zajíčková L., Eliáš M., Jašek O., Kudrle V., Frgala Z., Matějková J., Buršík J., Kadlečíková M. (2005). Atmospheric pressure microwave torch for synthesis of carbon nanotubes. Plasma Phys. Control. Fusion.

[45] Jašek O., Eliáš M., Zajíčková L., Kudrle V., Bublan M., Matějková J., Rek A., Buršík J., Kadlečíková M. (2006). Carbon nanotubes synthesis in microwave plasma torch at atmospheric pressure. Mater. Sci. Eng. C.

[46] Jašek O., Eliáš M., Zajíčková L., Kučerová Z., Matějková J., Rek A., Buršík J. (2007). Discussion of important factors in deposition of carbon nanotubes by atmospheric pressure microwave plasma torch. J. Phys. Chem. Solids.

[47] Zajíčková L., Eliáš M., Jašek O., Kučerová Z., Synek P., Matějková J., Kadlečíková M., Klementová M., Buršík J., Vojáčková A. (2007). Characterization of Carbon Nanotubes Deposited in Microwave Torch at Atmospheric Pressure. Plasma Process. Polym..

[48] Zajíčková L., Synek P., Jašek O., Eliáš M., David B., Buršík J., Pizurová N., Hanzlíková R., Lazar L. (2009). Synthesis of carbon nanotubes and iron oxide nanoparticles in MW plasma torch with Fe(CO)_5_ in gas feed. Appl. Surf. Sci..

[49] Pekárek J., Vrba R., Prášek J., Jašek O., Majzlíková P., Pekárková J., Zajíčková L. (2015). MEMS Carbon Nanotubes Field Emission Pressure Sensor with Simplified Design: Performance and Field Emission Properties Study. IEEE Sens. J..

[50] Synek P., Jašek O., Zajíčková L., David B., Kudrle V., Pizurová N. (2011). Plasmachemical synthesis of maghemite nanoparticles in atmospheric pressure microwave torch. Mater. Lett..

[51] Synek P., Jašek O., Zajíčková L. (2014). Study of Microwave Torch Plasmachemical Synthesis of Iron Oxide Nanoparticles Focused on the Analysis of Phase Composition. Plasma Chem. Plasma Process..

[52] Bhan R.K., Saxena R.S., Jalwania C.R., Lomash S.K. (2009). Uncooled Infrared Microbolometer Arrays and their Characterisation Techniques. Def. Sci. J..

[53] Majzlíková P., Prášek J., Eliáš M., Jašek O., Pekárek J., Hubálek J., Zajíčková L. (2014). Comparison of different modifications of screen-printed working electrodes of electrochemical sensors using carbon nanotubes and plasma treatment. Phys. Status Solidi.

[54] Hoa N.D., van Quy N., Cho Y., Kim D. (2007). An ammonia gas sensor based on non-catalytically synthesized carbon nanotubes on an anodic aluminum oxide template. Sens. Actuators B Chem..

[55] Sidek R.M., Yusof F.A.M., Yasin F.M., Wagiran R., Ahmadun F. Electrical response of multi-walled carbon nanotubes to ammonia and carbon dioxide.

[56] Firouzi A., Sobri S., Yasin F.M., Ahmadun F. Synthesis of Carbon Nanotubes by Chemical Vapor Deposition and their Application for CO_2_ and CH_4_ Detection.

[57] Han J.W., Kim B., Li J., Meyyappan M. (2014). A carbon nanotube based ammonia sensor on cellulose paper. RSC Adv..

[58] Vikramaditya T., Sumithra K. (2014). Effect of Substitutionally Boron-Doped Single-Walled Semiconducting Zigzag Carbon Nanotubes on Ammonia Adsorption. J. Comput. Chem..

[59] Teerapanich P., Myint M.T.Z., Joseph C.M., Hornyak G.L., Dutta J. (2013). Development and Improvement of Carbon Nanotube-Based Ammonia Gas Sensors Using Ink-Jet Printed Interdigitated Electrodes. IEEE Trans. Nanotechnol..

[60] Van Hieu N., Dung N.Q., Tam P.D., Trung T., Chien N.D. (2009). Thin film polypyrrole/SWCNTs nanocomposites-based NH3 sensor operated at room temperature. Sens. Actuators B Chem..

[61] Cheng C.Y., Nakashima M., Teii K. (2012). Low threshold field emission from nanocrystalline diamond/carbon nanowall composite films. Diam. Relat. Mater..

[62] Obraztsov A.N., Zakhidov A.A., Volkov A.P., Lyashenko D.A. (2003). Non-classical electron field emission from carbon materials. Diam. Relat. Mater..

